# Supplementary material for: Novel Driver Strength Index highlights important cancer genes in TCGA PanCanAtlas patients
Source: PeerJ. 2022 Aug 11;10:e13860. doi: 10.7717/peerj.13860 (PMC9375969; doi:10.7717/peerj.13860)
Supplement: Supplemental Information 2 [file peerj-10-13860-s002.zip › COHORTS/genes plots/age/2021_8_16_14_9_distribution_cohorts_males_65-69.pdf]

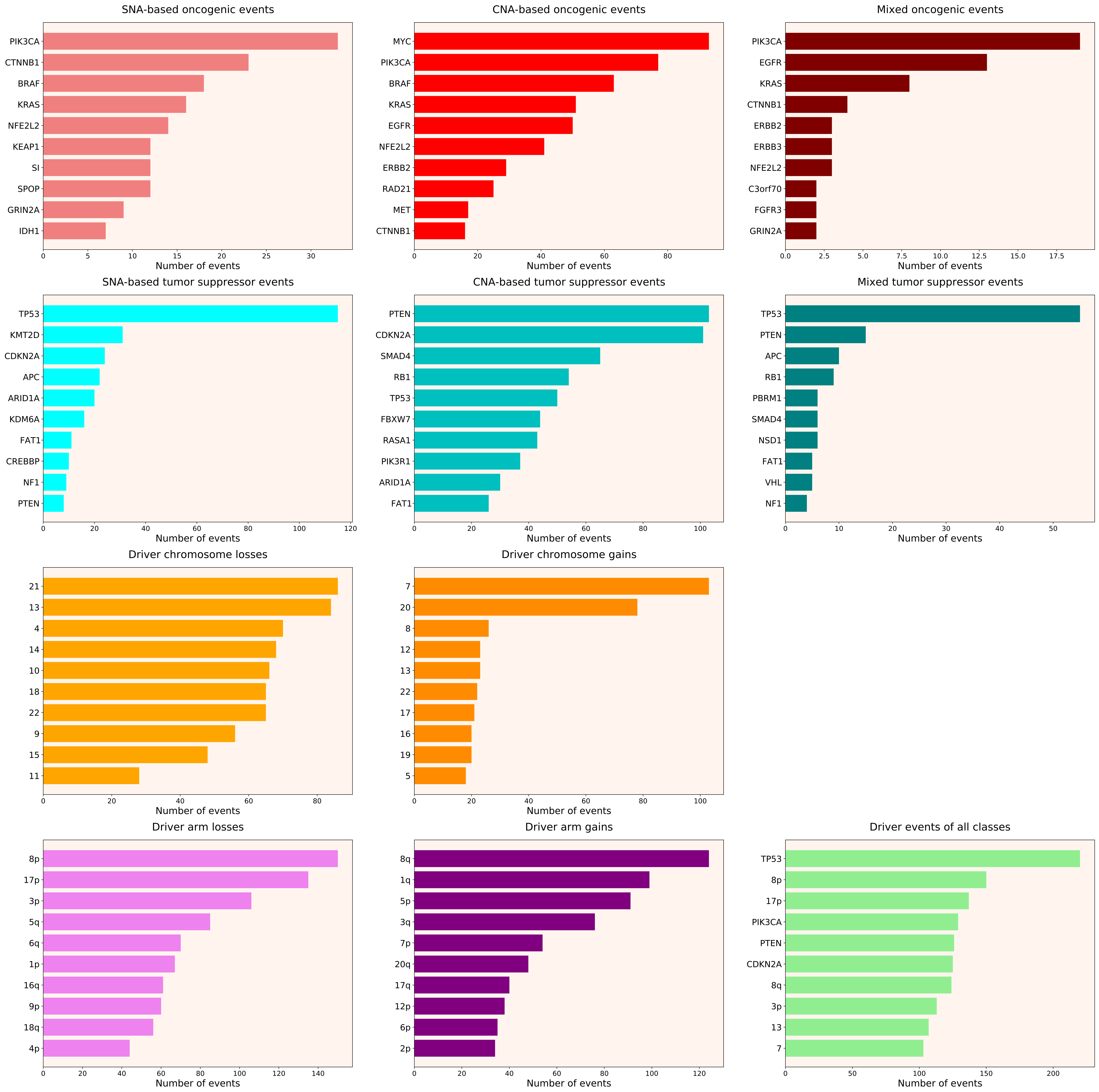
SNA-based oncogenic events
PIK3CA
CTNNB1
BRAF
KRAS
NFE2L2
KEAP1
SI
SPOP
GRIN2A
IDH1
0 5 10 15 20 25 30
Number of events
CNA-based oncogenic events
MYC
PIK3CA
BRAF
KRAS
EGFR
NFE2L2
ERBB2
RAD21
MET
CTNNB1
0 20 40 60 80
Number of events
Mixed oncogenic events
PIK3CA
EGFR
KRAS
CTNNB1
ERBB2
ERBB3
NFE2L2
C3orf70
FGFR3
GRIN2A
0.0 2.5 5.0 7.5 10.0 12.5 15.0 17.5
Number of events
SNA-based tumor suppressor events
TP53
KMT2D
CDKN2A
APC
ARID1A
KDM6A
FAT1
CREBBP
NF1
PTEN
0 20 40 60 80 100 120
Number of events
CNA-based tumor suppressor events
PTEN
CDKN2A
SMAD4
RB1
TP53
FBXW7
RASA1
PIK3R1
ARID1A
FAT1
0 20 40 60 80 100
Number of events
Mixed tumor suppressor events
TP53
PTEN
APC
RB1
PBRM1
SMAD4
NSD1
FAT1
VHL
NF1
0 10 20 30 40 50
Number of events
Driver chromosome losses
21
13
4
14
10
18
22
9
15
11
0 20 40 60 80
Number of events
Driver chromosome gains
7
20
8
12
13
22
17
16
19
5
0 20 40 60 80 100
Number of events
Driver arm losses
8p
17p
3p
5q
6q
1p
16q
9p
18q
4p
0 20 40 60 80 100 120 140
Number of events
Driver arm gains
8q
1q
5p
3q
7p
20q
17q
12p
6p
2p
0 20 40 60 80 100 120
Number of events
Driver events of all classes
TP53
8p
17p
PIK3CA
PTEN
CDKN2A
8q
3p
13
7
0 50 100 150 200
Number of events
